# The predictive validity of the Alcohol, Smoking and Substance Involvement Screening Test (ASSIST) for moderate‐ to high‐risk cannabis, methamphetamine and opioid use after release from prison

**DOI:** 10.1111/add.16138

**Published:** 2023-02-06

**Authors:** Craig Cumming, Stuart A. Kinner, Rebecca McKetin, Jesse T. Young, Ian Li, David B. Preen

**Affiliations:** ^1^ Centre for Health Services Research, School of Population and Global Health University of Western Australia Crawley WA Australia; ^2^ Centre for Adolescent Health Murdoch Children’s Research Institute Parkville VIC Australia; ^3^ Melbourne School of Population and Global Health University of Melbourne Parkville VIC Australia; ^4^ Griffith Criminology Institute Griffith University Mt Gravatt QLD Australia; ^5^ School of Population Health Curtin University Perth WA Australia; ^6^ National Drug and Alcohol Research Centre University of New South Wales Sydney NSW Australia; ^7^ Centre for Health Equity, Melbourne School of Population and Global Health University of Melbourne Parkville VIC Australia; ^8^ Centre for Adolescent Health Murdoch Children's Research Institute Parkville VIC Australia; ^9^ National Drug Research Institute Curtin University Perth WA Australia; ^10^ School of Population and Global Health University of Western Australia Crawley WA Australia

**Keywords:** Alcohol smoking and substance involvement tool, predictive validity, prison, risk, screening, substance use

## Abstract

**Background and aims:**

Illicit substance use is common among people entering prisons, as is returning to substance use after release from prison. We aimed to assess the predictive validity of the Alcohol, Smoking and Substance Involvement Screening Test (ASSIST) for returning to substance use after release from prison.

**Design:**

A longitudinal design with baseline survey conducted between 2008 and 2010 in the 6 weeks before expected prison release and up to three follow‐up surveys in the 6 months after release.

**Setting:**

Prisons in Queensland, Australia.

**Participants:**

A total of 1054 adults within 6 weeks of expected release from prison.

**Measurements:**

The ASSIST was used to assess problematic use of cannabis, methamphetamine, heroin and other non‐prescribed opioids in the 3 months before incarceration. Post‐incarceration substance use was measured at 1, 3 and 6 months after release. We calculated the area under the receiver operating characteristic curve (AUROC) and the optimal ASSIST cut‐off score for each substance, using Youden’s index (*J*).

**Findings:**

Forty‐one per cent (*n* = 434) of the cohort reported any substance use during follow‐up: 33% (*n* = 344) used cannabis, 20% (*n* = 209) methamphetamine, 10% (*n* = 109) heroin and 9% (*n* = 97) illicit other opioids. The optimal ASSIST cut‐off score was ≥ 4 for heroin, methamphetamine and cannabis and ≥ 1 for other opioids. Using these cut‐offs, the AUROC was highest for heroin in predicting both any use (AUROC = 0.82) and weekly use (AUROC = 0.88) in the past 4 weeks. AUROCs for other drugs ranged from 0.73 to 0.79.

**Conclusions:**

The ASSIST shows promise as an accurate and potentially scalable tool that may be useful for predicting a return to substance use after release from prison and could inform service delivery. The substantial rates of returning to substance use after release from prison suggest that prison serves to interrupt rather than cease substance use.

## INTRODUCTION

Rates of substance use are higher in people moving through prison compared to the general population. In the United States, the proportion of people in state prisons and jails meeting the criteria for a substance use disorder is approximately 12 times higher than the general population [1]. In Australia, the proportion of people entering prison with a history of substance use is approximately four times higher than that in the general population [2, 3]. The proportion of females entering Portuguese prisons with a history of amphetamine use was 84 times greater than the general population, while the proportion of males entering Portuguese prisons with a history of cocaine use was 29 times greater than the general population, both the highest of anywhere in Europe [4]. In Europe, cannabis, cocaine, amphetamines and heroin are the most prevalent illicit substances used by people entering European prisons [5], while among those entering prison in Australia the most prevalent illicit substances are cannabis, methamphetamine, heroin and non‐prescribed other opioids [2]. There is a range of systemic and structural disadvantages negatively impacting the health and wellbeing of this group [6, 7] that probably contribute to these high rates of substance use. Consequently, after release from prison, people face an elevated risk of substance‐related harms, including fatal [8, 9, 10] and non‐fatal overdose [11, 12], injury [13] and an increased risk of re‐incarceration [14, 15].

Despite this known risk, there is a substantial unmet need in most countries for alcohol and other drug (AOD) treatment services after release from prison [16, 17], and it is widely recognized that investment in prison throughcare is inadequate [18]. The evidence suggests that receiving AOD treatment services during incarceration [19], without treatment and support continuing after release from custody, is ineffective in preventing substance use and related harms after release. Therefore, prison probably serves as an interruption in the substance use trajectory of many people rather resulting in cessation of substance use [20, 21]. While drug policies continue to funnel people with problematic substance use into custodial settings [22, 23, 24], there is a need to provide continuous treatment and support both in custody and once people are released back into the community. Given the very limited resources currently available for AOD treatment throughcare, there is a clear imperative to identify individuals at high risk of returning to harmful substance use and target available resources to them.

The Alcohol, Smoking and Substance Involvement Screening Test (ASSIST) [25] screens for moderate and high‐risk substance use that may require treatment and has been shown to have good concurrent (produces similar results to other substance use screeners), construct (results correlate with measures of other outcomes believed to indicate substance use problems) and discriminative (results discriminate between participants with non‐problematic substance use, substance abuse or substance dependence) validity when tested on samples in community substance treatment and primary care settings [26, 27]. In prison settings, the ASSIST has demonstrated good test–retest reliability and is significantly correlated with the substance use disorder items of the Structured Clinical Interview for DSM‐IV‐non‐patient version with psychotic screen (SCID) [28, 29]. The ASSIST requires minimal training to administer, is brief and easy to score and can be administered electronically [29].

Although the ASSIST was developed as a screener for identifying people with recent, moderate‐ to high‐risk AOD use, given that substance dependence is often a chronically relapsing condition [30] it may also be suitable for identifying individuals at high risk of returning to substance use after release from prison. However, the predictive validity of the ASSIST for this purpose has not yet been established. This is particularly important given that substance use assessments conducted in prison often determine the provision of transitional and post‐release support, despite not being empirically evaluated for predictive ability.

Our aim was to assess the predictive validity of the ASSIST, administered in prison within 6 weeks of expected release, for returning to use of cannabis, methamphetamine, heroin and/or other opioids during the 6 months after release from prison.

## METHODS

### Study design

This prospective cohort study comprised a baseline survey of incarcerated men and women within 6 weeks of expected release and up to three follow‐up surveys in the 6 months after release from custody.

### Participants and procedure

Participants were initially recruited as part of a randomized trial of a case management intervention, described in more detail elsewhere [31, 32]. Briefly, 1325 adults were recruited within 6 weeks of their expected release from prisons in Queensland, Australia; all participants recruited between 2008 and 2010 were considered for this study. Of these, 1065 (80%) participants completed a baseline survey and at least one follow‐up survey within 6 months of release. Participants needed to have completed a minimum of one follow‐up survey to be included and did not need to have reported use of any of the substances being investigated. We used correctional records to identify date of reincarceration and release.

Participants were contacted approximately 1, 3 and 6 months after index prison release (first release after recruitment) to complete follow‐up surveys either in the community, or in prison if re‐incarcerated.

### Substance use measures

Substance use risk level for cannabis, methamphetamine, heroin and other non‐prescribed opioids during the 3 months before index incarceration (the period of incarceration during which they were recruited) was assessed retrospectively using the ASSIST [25], administered during the baseline survey within 6 weeks of expected release from prison. The ASSIST contains eight items, six of which are used to generate a total score with a possible range of 0–39 for each substance [25]. For each substance, raw ASSIST scores were categorized according to established cut‐offs into low‐/no‐risk (< 4), moderate‐risk (≥ 4 and < 27) or high‐risk (≥ 27) substance use before prison [25].

Substance use after release from prison was assessed at each follow‐up interview by asking participants if they had used each substance since either index release (for the 1‐month follow‐up survey) or the last follow‐up survey completed (for the 3‐ and 6‐month follow‐ups). Use of these brief items rather than the ASSIST during follow‐up was a pragmatic decision taken to accommodate inclusion of a number of other items in the follow‐up surveys. Participants who reported substance use were also asked on how many of the previous 28 days they had used each substance. Using these data we generated two binary outcome variables for each substance at each follow‐up time‐point: (1) any reported substance use and (2) at ‘least weekly substance use’ (i.e. use on ≥ 4 of the last 28 days).

### Other measures

We used the 10‐item Kessler psychological distress scale (K10) [33] to assess psychological distress at baseline. The K10 has a scoring range of 10–50. We dichotomized participants into two categories: low/no (≤ 15) and moderate/high [16, 17, 18, 19, 20, 21, 22, 23, 24, 25, 26, 27, 28, 29, 30, 31, 32, 33, 34, 35, 36, 37, 38, 39, 40, 41, 42, 43, 44, 45, 46, 47, 48, 49, 50] distress, consistent with the methodology used in the Australian National Health Survey [34]. We also used the seven‐item ENRICHD Social Support Inventory (ESSI) (scoring range = 8–34 with higher scores indicating greater social support) [35] to assess participants’ levels of access to social supports and support networks at baseline, which we dichotomized at the median (< 25 and 25+).

### Statistical analysis

All participant socio‐demographic characteristics (listed in Table 1) were dichotomized (see Supporting Information for details) and the proportion of participants with each characteristic was calculated. We tested for crude associations between socio‐demographic characteristics and returning to any use and weekly use of any of the four substances using univariable modified log‐linked Poisson regression with robust error variance, as recommended previously [36].

**TABLE 1:** Participant demographics as predictors of relapse to any and weekly use in the 6 months after release from prison.

	Any use		Weekly use		Total (%)
Baseline characteristics	RR (95% CI)	*P*	RR	*P*	
Age ≤ 25 years	1.18 (0.97, 1.44)	0.104	0.96 (0.73, 1.27)	0.795	309 (29)
Female	1.10 (0.88, 1.38)	0.394	1.27 (0.95, 1.68)	0.101	227 (22)
Indigenous	1.24 (1.00, 1.53)	0.054	0.99 (0.74, 1.34)	0.972	231 (22)
< 10 years schooling	1.27 (1.05, 1.53)	0.013	1.18 (0.92, 1.52)	0.182	442 (42)
Unemployed pre‐incarceration	1.61 (1.32, 1.96)	< 0.001	1.61 (1.24, 2.08)	< 0.001	547 (52)
Unstable housing pre‐incarceration	1.30 (1.05, 1.62)	0.019	1.14 (0.85, 1.54)	0.383	207 (20)
Not in stable relationship	1.15 (0.95, 1.40)	0.156	1.23 (0.95, 1.58)	0.120	614 (58)
Previous adult incarceration	2.26 (1.78, 2.86)	< 0.001	2.45 (1.78, 3.38)	< 0.001	681 (65)
Incarcerated as a juvenile	1.56 (1.28, 1.91)	< 0.001	1.31 (1.00, 1.71)	0.049	256 (24)
ESSI score < 25	1.14 (0.94, 1.38)	0.173	1.25 (0.98, 1.60)	0.076	489 (46)
Moderate/high distress (K10)	1.14 (0.95, 1.38)	0.163	1.50 (1.17, 1.93)	0.002	529 (50)
Current depression/anxiety	1.16 (0.92, 1.47)	0.216	1.55 (1.16, 2.07)	0.003	180 (17)

ESSI = ENRICHED Social Support Inventory; K10 = Kessler 10 measure of psychological distress; RR = relative risk; CI = confidence interval.

To assess the predictive validity of the ASSIST, we first collapsed participants’ responses across the three follow‐up surveys, such that anyone who reported use of a substance at any follow‐up was categorized as having used that substance during follow‐up, and anyone who reported at least weekly use of a substance at any follow‐up was categorized as having used that substance at least weekly during follow‐up.

We combined each individual’s self‐reported follow‐up substance use data for the four substances independently to create two substance use outcome variables for each substance: (1) a variable for returning to any use of each substance during follow‐up and (2) a variable for returning to at least weekly use of each substance during follow‐up.

Next, we calculated the area under the receiver operating characteristic (AUROC) curve and the associated optimal cut‐off score for each substance, both for any use and at least weekly use, using Youden’s index (*J*). This is a score that produces the maximum value of (sensitivity + specificity − 1), corresponding to the point farthest from chance on the curve [37].

Finally, for any and at least weekly use of each substance, we calculated the sensitivity, specificity, positive predictive value (PPV) and negative predictive value (NPV) of the ASSIST in predicting future self‐reported drug use, using both the established cut‐offs for moderate‐ and high‐risk use and the optimal cut‐offs we identified.

We assessed the internal consistency of the ASSIST at baseline, separately for each substance, by calculating Cronbach’s alpha [38].

To examine whether the length of index incarceration (and potential recall bias during administration of the ASSIST at baseline) had an impact on our AUROC, optimal scores, sensitivity and specificity, we performed sensitivity analyses. We restricted our analysis to participants with an index incarceration of less than 1 year. We also attempted subgroup analysis to see whether restricting our analysis to Indigenous participants, female participants and participants aged 25 years and under had an impact on our findings. We lacked sufficient statistical power to generate estimates for every drug at each cut‐off attempted; however, we report our partial results in Supporting information, Tables S3–S5, which should be interpreted with caution.

To investigate potential biased attrition, we also examined whether highest ASSIST score across all substances or other baseline variables (listed in Table 1) predicted loss to follow‐up, with participants in the low/no group used as the reference group. Please refer to the Supporting information for further details of our methods.

Our analysis was not pre‐registered, so all results should be considered exploratory. All statistical analyses were performed using Stata version 16 [39].

## RESULTS

Twenty‐nine per cent (*n* = 309) of participants were aged 25 years or less, 22% (*n* = 227) were female, 22% (*n* = 231) were Indigenous and 65% (*n* = 681) had previously been incarcerated as an adult (Table 1). Being unemployed in the 6 months prior to index incarceration was a crude predictor of returning to any use of any substance [relative risk (RR) = 1.61; 95% CI = 1.32, 1.96] and weekly use of any substance (RR = 1.61; 95% CI = 1.24, 2.08). Similarly, previous incarceration as an adult was a crude predictor of returning to use of any substance (RR = 2.26; 95% CI = 1.78, 2.86) and weekly use of any substance (RR = 2.45; 95% CI = 1.78, 3.38). Being incarcerated as a juvenile also crudely predicted returning to use of any substance (RR = 1.56; 95% CI = 1.28, 1.91) and weekly use of any substance (RR = 1.31; 95% CI = 1.00, 1.71).

### Follow‐up surveys

Follow‐up surveys were excluded from analysis if they were missing all data relating to use of all four substances being investigated (*n* = 64), occurred less than 28 days after index prison release (*n* = 23) or if the participant had spent no time in the community since their last follow‐up survey (*n* = 92). Exclusion of surveys from the analysis resulted in 11 participants being excluded from the study, leaving a final sample of 1054 participants (79% of total recruited). The 1054 participants completed their last interview a median of 189 days [interquartile range (IQR) = 182, 224; range = 29, 481] after index release. A total of 619 (59%) participants completed all three follow‐up surveys, 203 (19%) completed two and 232 (22%) completed one. Follow‐up surveys were completed by 844 (80%) participants at 1 month, 822 (78%) at 3 months and 829 (79%) at 6 months.

### Substance use

**FIGURE 1:**
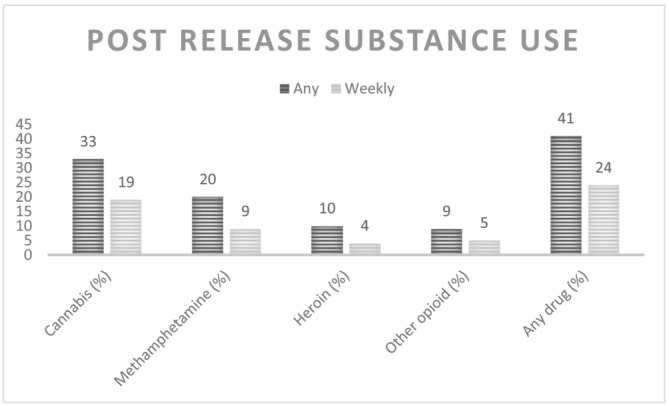
Proportion of participants with any substance use and at least weekly substance use (≥ 4 times in last 28 days) during follow‐up.

At baseline, the greatest proportion of participants screened for moderate‐risk use for cannabis (37%) and high‐risk use for methamphetamine (12%) using the ASSIST (Table 2). Cannabis (used by *n*=344 (33%) of participants) was the most common substance used after index release, followed by methamphetamine (*n*=209 (20%)), heroin (*n*=109 (10%)) and other unprescribed opioids (*n*=97 (9%)) (Fig. 1). Forty‐one per cent (*n*=434) of participants reported using at least one substance during follow‐up. A similar pattern emerged with respect to weekly substance use, with cannabis (*n*=195 (19%)) the most commonly reported, followed by methamphetamine (*n*=92 (9%)), other illicit opioids (*n*=48 (5%)) and heroin (*n*=48 (4%)). Overall, 24% (*n*=252) reported at least weekly use of at least one substance during follow‐up.

**TABLE 2:** Proportion of participants screening for moderate or high‐risk substance use at baseline using the Alcohol, Smoking and Substance Involvement Screening Test.

Substance	Moderate risk (%)	High risk (%)
Cannabis	386 (37)	98 (9)
Methamphetamine	279 (27)	131 (12)
Heroin	108 (10)	81 (8)
Other opioids	105 (10)	23 (2)

### ASSIST reliability and predictive validity

Cronbach’s alpha for the ASSIST at baseline was 0.85 for cannabis, 0.89 for illicit other opioids, 0.91 for methamphetamine and 0.95 for heroin. This suggests excellent internal consistency for the ASSIST for cannabis and illicit other opioids. However, some possible redundancy for the methamphetamine and heroin items [40], meaning fewer items for these substances, may be more optimal based on the results from our prison sample.

### Any substance use post‐release

For any instance of self‐reported substance use during the follow‐up period, all substances had an AUROC > 0.7 and < 0.9, indicating moderate accuracy [37, 41] (Table 3). The optimal ASSIST cut‐off score using *J* was 3 for all substances except illicit other opioids, which was 0. At the optimal cut‐off score, methamphetamine had the highest sensitivity (0.86) and other opioids had the lowest sensitivity (0.62); heroin had the highest specificity (0.89) and cannabis had the lowest specificity (0.69). At the optimal cut‐off score cannabis had the highest PPV (0.55), other opioids had the lowest PPV (0.34), heroin had the highest NPV (0.97) and cannabis had the lowest NPV (0.86).

**TABLE 3:** AUROC, optimal score thresholds for predicting any/weekly substance use during follow‐up post‐release and established ASSIST thresholds as predictors of any/weekly substance use during follow‐up post‐release.

	Optimal (Youden’s index)						Moderate risk (ASSIST ≥ 4)				High risk (ASSIST ≥ 27)			
Substance	Cut‐off	AUROC	Sens	Spec	PPV	NPV	Sens	Spec	PPV	NPV	Sens	Spec	PPV	NPV
Any use														
Cannabis	3	0.73	0.77	0.69	0.55	0.86	0.77	0.69	0.55	0.86	0.17	0.94	0.59	0.70
Methamphetamine	3	0.79	0.86	0.73	0.44	0.95	0.85	0.73	0.44	0.95	0.32	0.92	0.51	0.84
Heroin	3	0.82	0.76	0.89	0.44	0.97	0.75	0.89	0.44	0.97	0.38	0.96	0.51	0.93
Other opioids	0	0.75	0.62	0.87	0.34	0.96	0.52	0.92	0.40	0.95	0.09	0.99	0.39	0.91
Weekly use														
Cannabis	3	0.72	0.82	0.62	0.33	0.94	0.82	0.62	0.33	0.94	0.19	0.93	0.38	0.83
Methamphetamine	3	0.79	0.92	0.66	0.21	0.99	0.92	0.66	0.21	0.99	0.36	0.90	0.26	0.94
Heroin	3	0.88	0.91	0.86	0.22	0.99	0.91	0.86	0.22	0.99	0.49	0.94	0.28	0.98
Other opioids	0	0.77	0.69	0.86	0.20	0.98	0.63	0.91	0.24	0.98	0.13	0.98	0.27	0.96

ASSIST = Alcohol, Smoking and Substance Involvement Screening Test; AUROC = area under the receiver operating characteristic curve; NPV = negative predictive value; Sens = sensitivity; Spec = specificity; PPV = positive predictive value.

### Weekly substance use post‐release

For weekly substance use, heroin had the highest AUROC (0.88), followed by methamphetamine (0.79), other opioids (0.77) and cannabis (0.72). The optimal ASSIST cut‐off score using *J* was 3 for cannabis, methamphetamine and heroin and 0 for other opioids. At the optimal cut‐off score, methamphetamine had the highest sensitivity (0.92) and other opioids had the lowest sensitivity (0.69); heroin and other opioids had the highest specificity (0.86) and cannabis the lowest (0.62). At the optimal cut‐off, cannabis had the highest PPV (0.33), other opioids had the lowest PPV (0.20), methamphetamine and heroin had the highest NPV (0.99) and cannabis had the lowest NPV (0.94).

### Loss to follow‐up and sensitivity analysis

There was no association between participants’ highest ASSIST risk category across all drugs and loss to follow‐up, with neither the moderate‐risk group (RR = 1.00; 95% CI = 0.78, 1.29) nor high‐risk group (RR = 1.01; 95% CI = 0.76, 1.34) differing in loss to follow‐up compared to the low/no‐risk group. Statistically significant baseline demographic characteristic predictors of loss to follow‐up (Supporting information, Table S1) were identifying as Indigenous (RR = 1.62; 95% CI = 1.30, 2.05), being incarcerated as a juvenile (RR = 1.59; 95% CI = 1.26, 2.01) and not being in a stable relationship (RR = 1.37; 95% CI = 1.08, 1.74). Being previously incarcerated as an adult was included in the final model, but was not a statistically significant predictor (RR = 1.31; 95% CI = 0.99, 1.73).

Our AUROC sensitivity analysis (restricted to participants with an index incarceration < 1 year) produced similar results to our main analysis (Supporting information, Table S2).

## DISCUSSION

The ASSIST was originally designed to assess individuals for problematic substance use in the last 3 months to help inform primary care practitioners when substance use interventions may be required [42]. Our study is the first, to our knowledge, to assess the accuracy of the ASSIST for predicting a return to substance use in a large group of people being released from prison. Our results suggest that the ASSIST, when used to measure pre‐incarceration substance use for cannabis, methamphetamine, heroin and other opioids, can be used to accurately predict returning to substance use in the 6 months after release from prison. We also found, consistent with previous research [8, 11, 20, 21, 43, 44, 45, 46], that a substantial number of individuals returned to substance use in the 6 months after release from prison.

Our result, showing an optimal ASSIST score for predicting a return to use of other opioids of 0 compared to 3 for heroin, is provocative. This could be partly due to differences in the availability of heroin compared to other opioids in the period after release from prison, as well as possible different use and risk patterns for those using heroin alone compared to those using other opioids or a combination of the two. Given that a return to opioid use after release from prison can have serious consequences [8, 11], further research into this is probably warranted.

Predicting a return to substance use is a key policy priority, with extensive literature investigating how factors such as impulsivity [47, 48], adverse childhood experiences [49] and mental illness [50] may predict returning to substance use. Additionally, research investigates how using neurobiological markers and magnetic resonance imaging may also help to predict returning to substance use [51]. While this research is important in improving our understanding of returning to substance use, the methods used are often invasive and expensive and cannot be widely implemented in custodial systems with limited resources. There is an urgent need for simple, practical and scalable tools to identify individuals at risk of returning to substance use after release from custodial settings. We found that the ASSIST can be accurately used to predict returning to substance use after release from prison and is relatively quick, easy and cost‐effective to administer [29, 52], and therefore potentially scalable for use in prison settings. Our findings strongly support the routine use of the ASSIST in prison to identify individuals who may benefit from targeted throughcare support to reduce the risk of substance‐related harm after release from prison.

The only other study we are aware of that has assessed a substance use screening tool to predict future substance use in a prison context used the same cohort as our study [46], and found that the alcohol use disorders identification test (AUDIT) [53] is similarly accurate for predicting a return to hazardous alcohol use in the 3 months after release from prison [46]. While our results show that PPV was relatively low across substances (ranging from 0.33 to 0.53) when compared to the AUDIT (0.72) [46], this is probably explained by the relatively lower prevalence of the use of each illicit substance compared to alcohol among prisoners in Australia [2], with low prevalence resulting in lower PPV for screening tests [54]. While the PPV results suggest that there is a chance that the ASSIST may produce some ‘false positive’ results (i.e. screening individuals as moderate to high risk who are actually at low/no risk of returning to substance use), the high NPV results across all substances indicate that the chances are very low that the ASSIST will produce ‘false negative’ results (screening individuals as low/no risk when they are actually moderate to high risk of returning to substance use). From a policy perspective, it is preferable to err on the side of offering a larger number of people in prison substance use support (and potentially including some who may not truly be at moderate to high risk), while ensuring that those truly at moderate to high risk are not overlooked. Our results suggest that the ASSIST strikes this balance well for use in custodial settings.

Currently, AOD treatment programmes are offered in Queensland prisons [55]; however, we observed large numbers of people relapsing to substance use after release from prison, consistent with previous research showing that imprisonment alone often only results in a temporary interruption and a rapid return to substance use after release from prison [8, 11, 20, 21, 43, 44, 45, 46]. This raises two important issues. First, prison alone appears to be a suboptimal solution for addressing the needs of people with substance use problems. Secondly, if people with substance use problems are going to be incarcerated, there needs to be far greater investment in post‐release treatment and support aimed at reducing the risk of returning to high‐risk substance use. As continuity of care is crucial to minimize the risk of returning to substance use during the period after release from prison [56], specialist substance treatment such as therapeutic communities and opioid maintenance therapies commencing during incarceration and post‐release aftercare support should be a priority [57, 58]. Those responsible for service design and implementation should also consider our findings that previous incarceration as an adult or juvenile, being unemployed and/or having unstable housing in the 6 months before incarceration and completing fewer than 10 years of schooling predicted returning to drug use in the 6 months after prison. Additionally, previous research found that past injecting substance use [20, 21] and having social networks that were involved in substance use [59] were also predictors of returning to substance use after release from prison. Ensuring that individuals most at risk are offered targeted AOD support is crucial to capitalize on prison‐initiated abstinence or reduction in substance use and reduce the risk of recidivism, given the well‐documented association between AOD use and reincarceration [14, 60, 61, 62, 63].

Our large, reasonably representative, sample for this type of cohort is a key strength and this study remains the largest of its kind globally, although we still had insufficient statistical power to perform some subgroup analyses. Using self‐report data on stigmatized behaviours such as substance use may sometimes result in biases such as social desirability and recall bias; however, we addressed recall bias by performing sensitivity analysis, and previous research has found that self‐report substance use data are generally valid and reliable [64]. We observed a 21% loss to follow‐up, but our analysis suggested that attrition was not biased by participants’ ASSIST risk level. There were modest associations between loss to follow‐up and juvenile incarceration, Indigenous status and not being in a stable relationship that did not meaningfully impact our results. Our assessment of substance use during follow‐up was limited to whether participants had used illicit substances and, if so, on how many of the last 28 days. We did not explicitly assess participants for at least weekly substance use during follow‐up; rather, we made an assumption that where a participant reported using on at least 4 of the last 28 days, this equated to at least weekly use. It is possible that participants reporting substance use on 4 or more of the last 28 days did not use each week, so we may have picked up some binge, rather than weekly, substance use. To address this limitation, future research assessing post‐release substance use should use the ASSIST during follow‐up to capture richer data than simple self‐report items. Additionally, capturing data on the timing of substance use, using the time‐line follow‐back method [65, 66], could provide useful data regarding substance use patterns after release from prison.

Finally, our sample was recruited more than a decade ago. Changes in AOD treatment practice in Queensland prisons since study recruitment include the introduction of medication‐assisted treatment (MAT) for opioid dependence in some prisons; however, coverage remains poor, and ‘best practice’ injectable buprenorphine [67, 68] is not currently used, to the best of our knowledge. Given evidence that MAT has a modest protective effect, that rates of return to substance use after release from prison remain high even among those receiving MAT in prison [69, 70] and that the vast majority of our sample reported moderate/high‐risk use of substances other than opioids, we are confident that our findings remain relevant.

## CONCLUSION

A large number of people continue to return to substance use in the 6 months after release from prison. The ASSIST is a reliable and useful tool for ascertaining individuals at high risk of relapsing to substance use after release from prison. The ASSIST can be routinely used in prisons to ensure that throughcare AOD service resources are targeted towards individuals who need them the most, in an effort to reduce the risk of substance‐related harm after returning to the community. Substance treatment in prisons alone is demonstrably insufficient to prevent returning to risky substance use, and associated harms, after release from prison.

## DECLARATION OF INTERESTS

There are no competing interests.

## AUTHOR CONTRIBUTIONS

**Craig Cumming:** Conceptualization; data curation; formal analysis; investigation; methodology; project administration; software; writing ‐ original draft; writing ‐ review and editing. **Stuart A Kinner:** Conceptualization; data curation; funding acquisition; methodology; project administration; supervision; writing ‐ review and editing. **Rebecca McKetin:** Conceptualization; methodology; supervision; writing ‐ review and editing. **Jesse Tyler Young:** Conceptualization; data curation; methodology; software; writing ‐ review and editing. **Ian Li:** Supervision; writing ‐ review and editing. **David Brian Preen:** Conceptualization; data curation; funding acquisition; methodology; resources; supervision; writing ‐ review and editing.

## Supporting information


**Table S1.** Multivariable modified Poisson log‐linked regression with robust error variance model for predictors of loss to follow‐up
**Table S2.** AUROC and cutoff at optimal (Youden's index) score and validated ASSIST cutoff for predicting any and weekly self‐reported substance use during follow‐up for participants with index incarceration < 1 year
**Table S3.** AUROC and cutoff at optimal (Youden's index) score and validated ASSIST cutoff for predicting any and weekly self‐reported substance use during follow‐up for female participants only
**Table S4.** AUROC and cutoff at optimal (Youden's index) score and validated ASSIST cutoff for predicting any and weekly self‐reported substance use during follow‐up for participants age 25 years and under only
