# Supplementary material for: The predictive validity of the Alcohol, Smoking and Substance Involvement Screening Test (ASSIST) for moderate‐ to high‐risk cannabis, methamphetamine and opioid use after release from prison
Source: Addiction. 2023 Feb 6;118(6):1107–15. doi: 10.1111/add.16138 (PMC10952147; doi:10.1111/add.16138)
Supplement: Supplementary file 1 — Table S1. Multivariable modified Poisson log‐linked regression with robust error variance model for predictors of loss to follow‐up Table S2. AUROC and cutoff at optimal (Youden's index) score and validated ASSIST cutoff for predicting any and weekly self‐reported substance use during follow‐up for participants with index incarceration < 1 year Table S3. AUROC and cutoff at optimal (Youden's index) score and validated ASSIST cutoff for predicting any and weekly self‐reported substance use during follow‐up for female participants only Table S4. AUROC and cutoff at optimal (Youden's index) score and validated ASSIST cutoff for predicting any and weekly self‐reported substance use during follow‐up for participants age 25 years and under only [file ADD-118-1107-s001.docx]

## Loss to follow-up analysis

*Methods*

We measured potential baseline predictors of loss to follow-up using baseline survey items on demographic variables including age (dichotomised at median < 30 years vs ≥ 30 years), sex (female vs male), Indigenous status (Aboriginal/Torres Strait Islander/South Sea Islander vs non-Indigenous), years of schooling (< 10 years vs ≥ 10 years), employment status in the six months pre-index incarceration (unemployed vs employed full/part-time), housing status in the six months pre-index incarceration (unstable/no housing vs stable housing), current relationship status (in a stable relationship vs not), being incarcerated as an adult prior to index incarceration, being incarcerated as a juvenile, reporting a current diagnosis of depression or anxiety, and whether participants reported being likely to use each drug in the four weeks after release from prison. Additionally, participants completed the Enrichd Social Support Inventory (ESSI) (1); a self-report measure of social support. We dichotomised participants based on their ESSI median score (the median score was 25, we classified participants as either <25 or ≥25). We measured psychological distress using the Kessler Psychological Distress Scale (K10) (2) and dichotomised the participants based on level of distress reported (no/low distress vs moderate/high/very high distress). Starting with all sociodemographic variables in the model, we used backwards stepwise multivariable modified log-linked Poisson regression with robust error variance with the significance level set to 0.1 for removal.

We also investigated whether a participant's highest ASSIST risk category across all drugs was associated with being lost to follow-up (resulting in biased attrition). We assigned each participant a single risk category based on the highest ASSIST risk category that they screened for on any of the four drugs. We used univariable log-linked Poisson regression to calculate the relative risk (RR) for attrition for each risk group compared to the low/no risk group.

*Results*

In final multivariable modified Poisson model, statistically significant baseline demographic characteristic predictors of loss to follow-up (Table S1) were identifying as Indigenous (RR=1.62; 95%CI:1.30, 2.05), being incarcerated as a juvenile (RR=1.59; 95%CI:1.26, 2.01), not being in a stable relationship (RR=1.37; 95%CI:1.08,1.74). Being previously incarcerated as an adult was included in the final model, but was not a statistically significant predictor (RR=1.31; 95%CI:0.99,1.73).

There was no association between participants' highest ASSIST risk category across all drugs and loss to follow-up with either the moderate-risk group (RR=1.00; 95%CI:0.78, 1.29) or high-risk group (RR=1.01; 95%CI:0.76, 1.34), compared with the low/no risk group.

Table S1. Multivariable modified Poisson log-linked regression with robust error variance model for predictors of loss to follow-up

| Baseline characteristics | RR (95%CI) | P-value |
| --- | --- | --- |
| Identifying as Indigenous | 1.62 (1.30, 2.05) | **<0.001** |
| Not in a stable relationship | 1.37 (1.08, 1.74) | **0.010** |
| Previous adult incarceration | 1.31 (0.99, 1.73) | **0.058** |
| Juvenile incarceration | 1.59 (1.26, 2.01) | **<0.001** |

Table S2. AUROC and cutoff at optimal (Youden's index) score and validated ASSIST cutoff for predicting any and weekly self-reported substance use during follow-up for participants with index incarceration < 1 year

|  | Optimal (Youden's index) | | | | | | Moderate risk (ASSIST ≥ 4) | | | | High risk (ASSIST ≥ 27) | | | |
| --- | --- | --- | --- | --- | --- | --- | --- | --- | --- | --- | --- | --- | --- | --- |
| Substance | **Cut-off** | **AUROC** | **Sens** | **Spec** | **PPV** | **NPV** | **Sens** | **Spec** | **PPV** | **NPV** | **Sens** | **Spec** | **PPV** | **NPV** |
| Any use | | | | | | | | | | | | | | |
| Cannabis | 3 | 0.74 | 0.77 | 0.71 | 0.58 | 0.85 | 0.77 | 0.71 | 0.58 | 0.85 | 0.16 | 0.94 | 0.61 | 0.68 |
| Methamphetamine | 3 | 0.79 | 0.85 | 0.74 | 0.46 | 0.95 | 0.85 | 0.74 | 0.46 | 0.95 | 0.32 | 0.93 | 0.53 | 0.84 |
| Heroin | 3 | 0.82 | 0.75 | 0.89 | 0.43 | 0.97 | 0.75 | 0.89 | 0.43 | 0.97 | 0.34 | 0.96 | 0.48 | 0.93 |
| Other opioids | 0 | 0.79 | 0.70 | 0.88 | 0.35 | 0.97 | 0.59 | 0.92 | 0.41 | 0.96 | 0.11 | 0.98 | 0.40 | 0.92 |
| Weekly use | | | | | | | | | | | | | | |
| Cannabis | 3 | 0.72 | 0.82 | 0.63 | 0.34 | 0.94 | 0.82 | 0.63 | 0.34 | 0.94 | 0.17 | 0.93 | 0.34 | 0.83 |
| Methamphetamine | 3 | 0.79 | 0.91 | 0.67 | 0.22 | 0.99 | 0.91 | 0.67 | 0.22 | 0.99 | 0.36 | 0.90 | 0.28 | 0.93 |
| Heroin | 5 | 0.91 | 0.94 | 0.87 | 0.22 | 1.00 | 0.94 | 0.86 | 0.22 | 1.00 | 0.48 | 0.95 | 0.28 | 0.98 |
| Other opioids | 0 | 0.82 | 0.78 | 0.86 | 0.19 | 0.99 | 0.69 | 0.90 | 0.24 | 0.99 | 0.14 | 0.98 | 0.26 | 0.96 |

AUROC: area under the receiver operating characteristic curve; Sens: sensitivity; Spec: specificity PPV: positive predictive value

NPV: negative predictive value; ASSIST: Alcohol Smoking and Substance Involvement Screening Test

Table S3. AUROC and cutoff at optimal (Youden's index) score and validated ASSIST cutoff for predicting any and weekly self-reported substance use during follow-up for female participants only

|  | Optimal (Youden's index) | | | | | | Moderate risk (ASSIST ≥ 4) | | | | High risk (ASSIST ≥ 27) | | | |
| --- | --- | --- | --- | --- | --- | --- | --- | --- | --- | --- | --- | --- | --- | --- |
| Drug | **Cut-off** | **AUROC** | **Se** | **Sp** | **PPV** | **NPV** | **Se** | **Sp** | **PPV** | **NPV** | **Se** | **Sp** | **PPV** | **NPV** |
| Any use | | | | | | | | | | | | | | |
| Cannabis | 3 | 0.77 | 0.77 | 0.76 | 0.61 | 0.87 | 0.77 | 0.76 | 0.61 | 0.87 | - | - | - | - |
| Methamphetamine | 8 | 0.80 | 0.85 | 0.75 | 0.52 | 0.93 | - | - | - | - | 0.44 | 0.91 | 0.62 | 0.83 |
| Heroin | 0 | 0.86 | 0.92 | 0.81 | 0.37 | 0.99 | 0.84 | 0.85 | 0.41 | 0.98 | 0.36 | 0.96 | 0.50 | 0.92 |
| Other opioid | 0 | 0.80 | 0.74 | 0.86 | 0.32 | 0.97 | 0.53 | 0.90 | 0.33 | 0.95 | 0.05 | 0.98 | 0.20 | 0.92 |
| Weekly use | | | | | | | | | | | | | | |
| Cannabis | 3 | 0.75 | 0.81 | 0.69 | 0.41 | 0.93 | 0.81 | 0.69 | 0.41 | 0.93 | - | - | - | - |
| Methamphetamine | 5 | 0.78 | 0.94 | 0.62 | 0.29 | 0.98 | - | - | - | - | 0.47 | 0.88 | 0.38 | 0.91 |
| Heroin | 5 | 0.91 | 1.00 | 0.83 | 0.23 | 1.00 | 1.00 | 0.81 | 0.22 | 1.00 | 0.64 | 0.95 | 0.39 | 0.98 |
| Other opioid | 2 | 0.85 | 0.86 | 0.84 | 0.15 | 0.99 | 0.71 | 0.89 | 0.17 | 0.99 | 0.00 | 0.98 | 0.00 | 0.97 |

AUROC: area under the receiver operating characteristic curve; Sens: sensitivity; Spec: specificity PPV: positive predictive value

NPV: negative predictive value; ASSIST: Alcohol Smoking and Substance Involvement Screening Test

Table S4. AUROC and cutoff at optimal (Youden's index) score and validated ASSIST cutoff for predicting any and weekly self-reported substance use during follow-up for participants age 25 years and under only

|  | Optimal (Youden's index) | | | | | | Moderate risk (ASSIST ≥ 4) | | | | High risk (ASSIST ≥ 27) | | | |
| --- | --- | --- | --- | --- | --- | --- | --- | --- | --- | --- | --- | --- | --- | --- |
| Drug | Cut-off | AUROC | Se | Sp | PPV | NPV | Se | Sp | PPV | NPV | Se | Sp | PPV | NPV |
| Any use | | | | | | | | | | | | | | |
| Cannabis | 3 | 0.67 | 0.80 | 0.54 | 0.51 | 0.82 | 80 | 0.54 | 0.51 | 0.82 | 0.21 | 0.90 | 0.56 | 0.66 |
| Methamphetamine | 3 | 0.79 | 0.88 | 0.69 | 0.45 | 0.95 | 0.88 | 0.69 | 0.45 | 0.95 | 0.42 | 0.93 | 0.64 | 0.85 |
| Heroin | 2 | 0.81 | 0.74 | 0.88 | 0.40 | 0.97 | 0.71 | 0.90 | 0.44 | 0.97 | - | - | - | - |
| Other opioid | 0 | 0.72 | 0.57 | 0.88 | 0.33 | 0.95 | 0.47 | 0.92 | 0.39 | 0.94 | - | - | - | - |
| Weekly use | | | | | | | | | | | | | | |
| Cannabis | 10 | 0.68 | 0.72 | 0.64 | 0.29 | 0.92 | 0.85 | 0.46 | 0.25 | 0.94 | 0.28 | 0.89 | 0.35 | 0.86 |
| Methamphetamine | 3 | 0.79 | 0.96 | 0.61 | 0.19 | 0.99 | 0.96 | 0.61 | 0.19 | 0.99 | 0.46 | 0.89 | 0.28 | 0.95 |
| Heroin | 10 | 0.90 | 0.90 | 0.91 | 0.25 | 0.99 | 0.90 | 0.86 | 0.18 | 0.99 | - | - | - | - |
| Other opioid | 9 | 0.85 | 0.75 | 0.95 | 0.38 | 0.99 | 0.75 | 0.91 | 0.25 | 0.99 | - | - | - | - |

AUROC: area under the receiver operating characteristic curve; Sens: sensitivity; Spec: specificity PPV: positive predictive value

NPV: negative predictive value; ASSIST: Alcohol Smoking and Substance Involvement Screening Test

Table S4. AUROC and cutoff at optimal (Youden's index) score and validated ASSIST cutoff for predicting any and weekly self-reported substance use during follow-up for Indigenous Australian participants only

|  | Optimal (Youden's index) | | | | | | Moderate risk (ASSIST ≥ 4) | | | | High risk (ASSIST ≥ 27) | | | |
| --- | --- | --- | --- | --- | --- | --- | --- | --- | --- | --- | --- | --- | --- | --- |
| Drug | Cut-off | AUROC | Se | Sp | PPV | NPV | Se | Sp | PPV | NPV | Se | Sp | PPV | NPV |
| Any use | | | | | | | | | | | | | | |
| Cannabis | 5 | 0.70 | 0.72 | 0.67 | 0.61 | 0.77 | 0.74 | 0.63 | 0.60 | 0.77 | 0.25 | 0.91 | 0.67 | 0.63 |
| Methamphetamine | 2 | 0.84 | 0.90 | 0.77 | 0.47 | 0.97 | - | - | - | - | 0.31 | 0.95 | 0.57 | 0.85 |
| Heroin | 3 | 0.79 | 0.65 | 0.94 | 0.54 | 0.96 | 0.65 | 0.94 | 0.54 | 0.96 | - | - | - | - |
| Other opioid | 2 | 0.77 | 0.59 | 0.96 | 0.50 | 0.96 | 0.55 | 0.96 | 0.60 | 0.95 | 0.14 | 0.99 | 0.60 | 0.92 |
| Weekly use | | | | | | | | | | | | | | |
| Cannabis | 5 | 0.68 | 0.78 | 0.58 | 0.32 | 0.91 | 0.80 | 0.54 | 0.31 | 0.92 | 0.28 | 0.88 | 0.36 | 0.83 |
| Methamphetamine | 2 | 0.85 | 1.00 | 0.69 | 0.19 | 1.00 | - | - | - | - | 0.27 | 0.91 | 0.17 | 0.95 |
| Heroin | 10 | 0.84 | 0.75 | 0.92 | 0.15 | 0.99 | 0.75 | 0.89 | 0.11 | 0.99 | - | - | - | - |
| Other opioid | 11 | 0.89 | 0.80 | 0.98 | 0.62 | 0.99 | 0.80 | 0.95 | 0.40 | 0.99 | 0.20 | 0.99 | 0.40 | 0.96 |

AUROC: area under the receiver operating characteristic curve; Sens: sensitivity; Spec: specificity PPV: positive predictive value

NPV: negative predictive value; ASSIST: Alcohol Smoking and Substance Involvement Screening Test

References

1. Mitchell P. H., Powell L., Blumenthal J., Norten J., Ironson G., Pitula C. R. et al. A short social support measure for patients recovering from myocardial infarction: the ENRICHD Social Support Inventory. *Journal of Cardiopulmonary Rehabilitation and Prevention* 2003: 23: 398-403.

2. Andrews G., Slade T. Interpreting scores on the Kessler psychological distress scale (K10). *Australian and New Zealand Journal of Public Health* 2001: 25: 494-497.
